# Supplementary material for: The final step of 40S ribosomal subunit maturation is controlled by a dual key lock
Source: eLife. 2021 Apr 28;10:e61254. doi: 10.7554/eLife.61254 (PMC8112863; doi:10.7554/eLife.61254)
Supplement: Supplementary file 1. [file elife-61254-supp1.docx]

| **Supplementary File 1.** Cryo-EM data collection, atomic models refinement and validation statistics. | | | | | | | | | |
| --- | --- | --- | --- | --- | --- | --- | --- | --- | --- |
| Microscope model | FEI Titan Krios cryo-transmission electron microscope | | | | | | | | |
| Detector model | Gatan K2 summit direct electron detector | | | | | | | | |
| Number of datasets | 1 | | | | | | | | |
| Number of micrographs collected | 9,494 | | | | | | | | |
| Pixel size (Å) | 1.04 | | | | | | | | |
| Defocus range (μm) | 0.8 – 2.8 | | | | | | | | |
| Voltage (kV) | 300 | | | | | | | | |
| Electron dose (e^-^Å^-2^) | 29.88 | | | | | | | | |
| Name of  3D reconstruction/  model | | State A RIO1(kd)-StHA | State B RIO1(kd)-StHA | State A head | State A body | State A platform | State B head | State B body | State B platform |
| EMDB entry of map | | EMD- 11440 | EMD-11441 | EMD-11446 | EMD-11445 | EMD-11447 | EMD-11443 | EMD-11442 | EMD-11444 |
| PDB entry of the full model | | 6ZU0 | 6ZV6 |  |  |  |  |  |  |
| Final number of particles | | 104,844 | 276,012 | 104,844 | 104,844 | 104,844 | 276,012 | 276,012 | 276,012 |
| Resolution (Å) (FSC threshold = 0.143) | | 3.22 | 3.00 | 3.17 | 3.14 | 3.30 | 2.96 | 2.98 | 2.98 |
| Map sharpening B-factor (Å^2^) | | -89 | -88 | -68 | -71 | -81 | -84 | -80 | -77 |
| **Refinement and model validation statistics^(^**^a)^ | | | | | | | | | |
| Model refinement resolution range ( Å)  Model resolution (Å) (FSC threshold = 0.5)  Clashscore (all atoms) | | 40-2.7  3.61  7.53 | 40-2.6  3.52  6.72 |  |  |  |  |  |  |
| MolProbity Score  Protein | | 1.81 | 1.77 |  |  |  |  |  |  |
| Rmsd (bonds lengths, Å) | | 0.0084 | 0.01 |  |  |  |  |  |  |
| Rmsd (angles, °) | | 1.20 | 1.00 |  |  |  |  |  |  |
| Ramachandran plot (%)  favored | | 94.19 | 94.15 |  |  |  |  |  |  |
| allowed | | 5.73 | 5.73 |  |  |  |  |  |  |
| outliers | | 0.08 | 0.12 |  |  |  |  |  |  |
| RNA | |  | | | | | | | |
| Correct sugar puckers (%) | | 97.2 | 97.8 |  |  |  |  |  |  |
| Good backbone conformation (%) | | 69.4 | 73.7 |  |  |  |  |  |  |
| ^(a)^Models were validated using MolProbity implemented in PHENIX.REFINE (Adams et al., 2010) | | | | | | | | | |
