# Supplementary material for: The final step of 40S ribosomal subunit maturation is controlled by a dual key lock
Source: eLife. 2021 Apr 28;10:e61254. doi: 10.7554/eLife.61254 (PMC8112863; doi:10.7554/eLife.61254)
Supplement: Transparent reporting form [file elife-61254-transrepform.docx]

***eLife’s* transparent reporting form**

We encourage authors to provide detailed information *within their submission* to facilitate the interpretation and replication of experiments. Authors can upload supporting documentation to indicate the use of appropriate reporting guidelines for health-related research (see EQUATOR Network), life science research (see the [BioSharing Information Resource](https://biosharing.org/)), or the [ARRIVE guidelines](http://www.plosbiology.org/article/info:doi/10.1371/journal.pbio.1000412) for reporting work involving animal research. Where applicable, authors should refer to any relevant reporting standards documents in this form.

If you have any questions, please consult our Journal Policies and/or contact us: [editorial@elifesciences.org](mailto:editorial@elifesciences.org).

**Sample-size estimation**

- You should state whether an appropriate sample size was computed when the study was being designed
- You should state the statistical method of sample size computation and any required assumptions
- If no explicit power analysis was used, you should describe how you decided what sample (replicate) size (number) to use

Please outline where this information can be found within the submission (e.g., sections or figure legends), or explain why this information doesn’t apply to your submission:

No explicit power analysis was used to determine the sample size for each experiment. Sample size was based on empiric considerations taking into account both robustness of the respective assay and the experimental challenge of acquiring the data.

**Replicates**

- You should report how often each experiment was performed
- You should include a definition of biological versus technical replication
- The data obtained should be provided and sufficient information should be provided to indicate the number of independent biological and/or technical replicates
- If you encountered any outliers, you should describe how these were handled
- Criteria for exclusion/inclusion of data should be clearly stated
- High-throughput sequence data should be uploaded before submission, with a private link for reviewers provided (these are available from both GEO and ArrayExpress)

Cryo-EM experiments presented in Figures 2 and 3 were performed once. The number of recorded micrographs as well as particles in each map are indicated in the material and methods section, as well as in Supplementary Figure 3. All other experiments were performed in triplicates, except for Figure 6 which were performed five times. These repetitions refer to the number of times the entire experiment was performed on independent days and therefore correspond to biological replicates of the experiment.

**Statistical reporting**

- Statistical analysis methods should be described and justified
- Raw data should be presented in figures whenever informative to do so (typically when N per group is less than 10)
- For each experiment, you should identify the statistical tests used, exact values of N, definitions of center, methods of multiple test correction, and dispersion and precision measures (e.g., mean, median, SD, SEM, confidence intervals; and, for the major substantive results, a measure of effect size (e.g., Pearson's r, Cohen's d)
- Report exact p-values wherever possible alongside the summary statistics and 95% confidence intervals. These should be reported for all key questions and not only when the p-value is less than 0.05.

Please outline where this information can be found within the submission (e.g., sections or figure legends), or explain why this information doesn’t apply to your submission:

(For large datasets, or papers with a very large number of statistical tests, you may upload a single table file with tests, Ns, etc., with reference to sections in the manuscript.)

For experiments that were quantified, the number of replicates and precision measures (SD) can be found in the figure legends (Figs 4 and 5). Statistics were applied to analyze results in Figure 6 using a non-parametric test (Wilcoxon) on the medians. For mass spectrometry experiments, data analysis is described in the materials and methods section.

**Group allocation**

- Indicate how samples were allocated into experimental groups (in the case of clinical studies, please specify allocation to treatment method); if randomization was used, please also state if restricted randomization was applied
- Indicate if masking was used during group allocation, data collection and/or data analysis

Please outline where this information can be found within the submission (e.g., sections or figure legends), or explain why this information doesn’t apply to your submission:

N/A

**Additional data files (“source data”)**

- We encourage you to upload relevant additional data files, such as numerical data that are represented as a graph in a figure, or as a summary table
- Where provided, these should be in the most useful format, and they can be uploaded as “Source data” files linked to a main figure or table
- Include model definition files including the full list of parameters used
- Include code used for data analysis (e.g., R, MatLab)
- Avoid stating that data files are “available upon request”

Please indicate the figures or tables for which source data files have been provided:

The mass spectrometry proteomics data have been deposited to the ProteomeXchange Consortium via the PRIDE partner repository with the dataset identifier PXD019270.

Cryo-EM maps have been deposited in the Electron Microscopy Data Bank (EMDB), under the accession codes : EMD-11440 (State A multi-body composite map); EMD-11441 (State B multi-body composite map); EMD-11446 (State A, head); EMD-11445 (State A, body); EMD-X11447 (State A, platform); EMD-11443 (State B, head); EMD-11442 (State B, body); EMD-11444 (State B, platform). Atomic coordinate models of State A and State B RIO1(kd)-StHA pre-40S particles have been deposited in the Protein Data Bank (PDB), with respective PDB accession codes 6ZU0 and 6ZV6.
